# Another crack in the Dark Taxa wall: a custom DNA barcoding protocol for the species-rich and common Eurytomidae (Hymenoptera, Chalcidoidea)

**DOI:** 10.3897/BDJ.11.e101998

**Published:** 2023-05-09

**Authors:** Samin Jafari, Björn Müller, Björn Rulik, Vera Rduch, Ralph S. Peters

**Affiliations:** 1 Leibniz Institute for the Analysis of Biodiversity Change (LIB), Zoological Research Museum Alexander Koenig (zfmk), Arthropoda Department, Adenauerallee 127, D-53113, Bonn, Germany Leibniz Institute for the Analysis of Biodiversity Change (LIB), Zoological Research Museum Alexander Koenig (zfmk), Arthropoda Department, Adenauerallee 127, D-53113 Bonn Germany

**Keywords:** GBOLIII: Dark Taxa, barcoding, Eurytomidae, COI, PCR, COI primer

## Abstract

DNA barcodes are a great tool for accelerated species identification and for complementing species delimitation. Furthermore, DNA barcode reference libraries are the decisive backbone feature for any metabarcoding study in biodiversity monitoring, conservation or ecology. However, in some taxa, DNA barcodes cannot be generated with published primers at a satisfying success rate and these groups will consequently be largely missing from any barcoding-based species list. Here, we provide a custom DNA barcoding forward primer for the Eurytomidae (Hymenoptera, Chalcidoidea), elevating the success rate of high-quality DNA barcodes from 33% to 88%. Eurytomidae is a severely understudied, taxonomically challenging, species-rich group of primarily parasitoid wasps. High species numbers, diverse ecological roles and widespread and common presence identify Eurytomidae as one of many crucial families in terrestrial ecosystems. It is now possible to include Eurytomidae when studying and monitoring the terrestrial fauna, highlighting that barcoding-based approaches will need to routinely use different primers to avoid biases in their data and inferences. The new DNA barcoding protocol is also a prerequisite for our integrative taxonomy study of the group, aiming at delimiting and characterising Central European species and filling the GBOL (German Barcode Of Life) DNA barcode reference library with species-named and voucher-linked sequences.

## Introduction

Eurytomidae is one of the largest families of Chalcidoidea, with over 1400 described species in 97 genera (Aguiar et al. 2013; Noyes 2019). For Germany, where our research is based as part of the GBOL III: Dark Taxa project targeting “dark” insect taxa in Central Europe (https://bolgermany.de/home/gbol3/projects/, Hausmann et al. (2020)), 101 species have been recorded (Vidal et al. 2022). Eurytomids exhibit an extraordinarily wide variety of life histories, with many being parasitoids of various insect taxa and life stages and others being secondarily phytophagous as seed eaters, stem feeders or gall inducers, associated with various plant taxa (Lotfalizadeh et al. 2007a). Like many parasitoid wasps, eurytomids have a small body size, and species are comparatively monotonous in appearance, difficult to separate morphologically and, ergo,the family is still harbouring many taxonomic uncertainties (Lotfalizadeh et al. 2007a, Gebiola et al. 2012, Claridge and Askew 1960). Despite being abundantly trapped in biodiversity surveys (Noyes and Valentine 1989) and long recognised as ecologically diverse and species-rich, their ecological importance is probably still underestimated and their presence in biodiversity monitoring samples is uncategorised. They are prime examples of so-called “Dark Taxa” for which even basic data on taxonomy and life history are missing and for which reference collections, meaningful checklists and taxonomic specialists, as well as DNA barcode data in public reference databases, are widely absent. For example, only 107 out of over 1400 species of Eurytomidae are present with named DNA barcodes in BOLD (http://boldsystems.org/index.php/Taxbrowser_Taxonpage?taxon=eurytomidae&searchTax=Search+Taxonomy, accessed 30/11/2022). DNA barcodes have often been shown to be a highly useful and reliable tool for species identification (Hebert et al. 2004a, Hebert et al. 2004b, Barrett and Hebert 2005, Ward et al. 2005, Clare et al. 2007, Smith et al. 2006, Smith et al. 2007, Seifert et al. 2007) and also for species delimitation when implemented in an integrative taxonomy framework (Chen et al. 2004, Lotfalizadeh et al. 2007b, Yanwei et al. 2009, Zhang et al. 2013, Zhang 2013, Delvare et al. 2014, Delvare et al. 2019). Large consortia have been filling DNA barcode reference libraries for application in, for example, barcoding and metabarcoding-based biodiversity monitoring, ecology or conservation efforts (Zizka et al. 2022). Ideally, all species present in analysed samples will match against a well-curated database entry and underlying studies will be able to work with the full picture of complete species lists to get to the best possible inferences. While impressive progress has already been made, we are aware that, currently, this is not the case. Barcode reference libraries are still very much incomplete (Hausmann et al. 2020), taxonomic issues are countless even in comparatively well-studied faunae (Hausmann et al. 2020), metabarcoding suffers from methodological issues (Zizka et al. 2022) and standard primers will not bind to the DNA of many species-rich taxa, i.e. standard protocols will gain only low success rates (Vasilita et al. 2022).

In this study, we show that DNA barcoding of Eurytomidae with standard primers will yield only very limited data and that, with a modified protocol and newly-designed primer, DNA barcode data can be generated at a high success rate. Additionally, we briefly discuss the importance of routinely using different primers when targeting terrestrial fauna with DNA (meta)-barcoding and also highlight the importance of large-scale DNA barcode data for integrative taxonomy in the Eurytomidae.

## Material and methods

### Sampling

We started with the first batch of 190 Eurytomidae specimens, available from the material assembled for the GBOL III: Dark Taxa project. This first test batch was optimised to include several relevant Eurytomidae genera present in Germany to cover a broad taxonomic range, samples from different regions of Germany to cover the geographic target country of the project and samples of different age to realistically represent the samples we had at hand. All samples were registered in our sample database, identified to genus level and stored in 96% ethanol at 4°C or less.

The samples included the Eurytomidae genera *Eurytoma*, *Sycophila* and *Tetramesa*. They originate from Hesse (77), Bavaria (55), Baden-Wuerttemberg (43) and Schleswig-Holstein (2). Samples were collected over a period of 12 years, starting in August 2009 and ending in June 2021. Specimens were collected with sweep nets, Malaise traps, canopy fogging or with suction traps.

**Figure 1.**
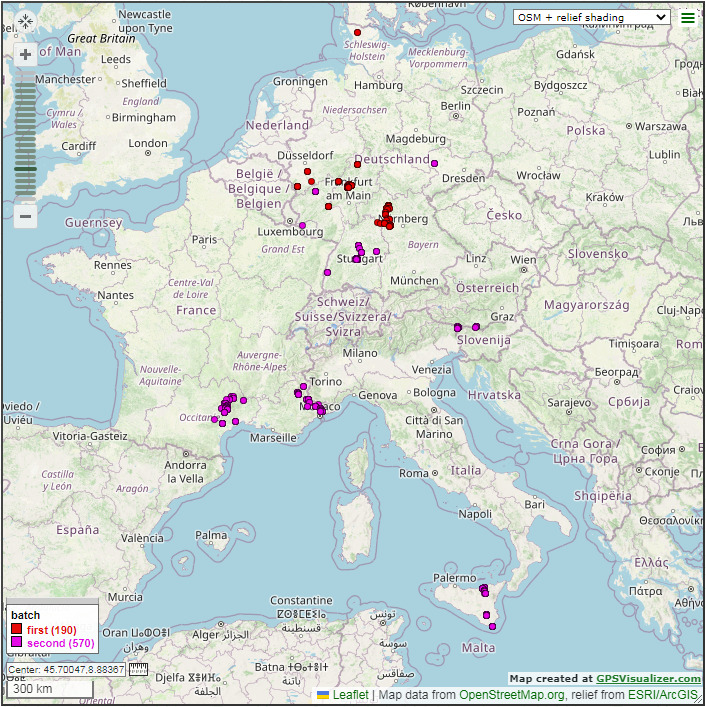
Map of collection sites of samples used in Eurytomidae barcoding. The 26 sampling sites from the first batch are marked in red. Samples were collected between August 2009 and June 2021. The 112 sites from the second batch are marked in purple. Samples were collected between July 2008 and June 2021. Some of the sites are too close to each other and, therefore, cannot be shown as individual points.

A second batch of additional 570 samples (making a total of 760 samples) was processed specifically with the newly-designed primer for an extended check of the achieved success rate, including samples from a broader taxonomic and geographic range. A total of 212 samples of the second batch came from Germany, eight from Austria, 253 from France and 97 from Italy (Fig. 1). The collection period stretched from July 2008 to July 2021. The samples include the genera *Bruchophagus*, *Eurytoma* and *Eurytomocharis*.

### DNA extraction, PCR and PCR conditions

DNA extraction was performed using the magnetic bead-based BioSprint 96 DNA Blood Kit (QIAGEN GmbH - Germany). Accordingly, lysis and DNA extraction were performed individually for each animal in a 12 x 8 plate format. Each animal was lysed separately and non-destructively in 180 µl ATL buffer and 20 µl proteinase K for 12-14 hours at 56°C with permanent shaking at 300 rpm (Eppendorf Thermomixer^®^ comfort). After lysis, the animals are removed from the DNA containing ATL buffer for later card mounting. The magnetic bead master mix consists of 22 ml AL buffer, 22 ml isopropanol and 3.2 ml MagAttract. From this mixture, 450 µl are pipetted into each ATL sample. Now, the DNA is washed in five steps: 1) adding 650 µl AW1 buffer, 2) adding 500 µl AW1, 3) and 4) adding 500 µl AW2 each, 5) adding 500 µl DNAse-free water plus TWEEN^®^ (Sigma-Aldrich^®^). The washed DNA is finally eluted in 200 µl AE buffer. Washing and DNA-elution is done by BioSprint 96 Purification System (Thermo Scientific/QIAGEN).

PCR was done with the QIAGEN Multiplex PCR Kit. The master mix for a 96 PCR well plates is composed as follows: 1000 µl Multiplex, 200 µl Q-Solution, 440 µl RNase-free water and 80 µl of each forward and reverse primer (10 pmol/µl). Each well is filled with 18 µl PCR Mastermix and 2 µl DNA from the elution plate.

The conditions (using GeneAmp^®^ PCR System 9700) for touchdown PCR (Astrin and Stüben 2008) are as follows: Initial 95°C for 15 minutes, followed by 94°C denaturation for 35 seconds, 55°C annealing for 90 seconds and 72°C elongation for 90 seconds. Denaturation, annealing and elongation are repeated 15 times, with the annealing temperature decreasing by 1°C in each cycle. When the annealing temperature reaches 40°C, there is no further reduction in temperature. Denaturation, annealing and elongation are repeated 25 times as described above. The PCR ends with a final elongation at 72°C for 10 minutes and is then cooled to 12°C permanently.

### Sequencing and sequence analysis

Sanger sequencing of COI-PCR products was carried out by BGI BIO Solutions Co, Ltd (Hong Kong). The analysis of the sequence data was carried out with Geneious v. 7.1.9 (http://www.geneious.com). The sequences were classified into four quality categories: 1) High, 2) Medium, 3) Low quality and 4) failed sequencing. High quality sequences were those that match the GBOL Gold Standard, i.e. the peak quality of the raw sequence data (chromatogram), respectively, the quality of the final consensus sequence, were classified as quality level High by Geneious and disagreements and ambiguities within the consensus sequence were ≤ 1%. Consensus sequences classified as Medium or Low quality by Geneious did not match the Gold Standard even if the disagreements and ambiguities were equal or less than 1%. Consensus sequences classified as High, but with more than 1% disagreements and ambiguities also did not match the GBOL Gold Standard.

### Primer, sequence alignment and primer design

The standard primers used in GBOL III (at the ZFMK) for COI barcoding of insect samples are LCO 1490-JJ/HCO 2198-JJ (Astrin and Stüben 2008) and this was used in the first round of processing the batch of 190 Eurytomidae samples. When it became apparent that the success rate was unsatisfying (i.e. far below 80%, see results), we were looking for alternatives for the forward primer (the standard reverse primer HCO 2198-JJ can be used also in Eurytomidae). Based on a sequence alignment including those samples which were successfully sequenced, we first checked whether other published (forward) primers will potentially yield better results. LCO 1490 (Folmer et al. 1994) or LepF (Hajibabaei et al. 2006) target the same binding site as LCO 1490-JJ and are largely similar in sequence and alignment checks indicate clearly that also these primers will not bind at the satisfying rate (Suppl. material 1). Then, we scanned available eurytomid COI long read sequences with hyden (Linhart and Shamir 2002) for alternative binding sites around the forward tip of the Folmer primer region, but without success. Finally, based on the sequence alignment, we created a new forward primer sequence **dEURYT-BR1** (Table 1, Suppl. material 2 and Fig. 3) and re-processed the 190 samples. Based on the alignment of those samples which were successfully sequenced with **dEURYT-BR1**, we again adjusted the forward primer sequence for the final primer **dEURYT-BRBM2** (Suppl. material 3, Table 1 and Fig. 3). Again, the 190 samples were re-processed, now using the **dEURYT-BRBM2** forward primer.

As already mentioned, the reverse primer HCO 2198-JJ works for the Eurytomidae. Accordingly, it would be advantageous if the newly-designed forward primer could be adapted to the existing PCR conditions. However, a melting temperature adjusted between forward and reverse primer should not, for example, be at the expense of dimer formation, which would impair PCR effectiveness (Apte and Daniel 2009). Therefore, GC content of new primer sequences, melting temperature (°C), self-dimer check, base composition, Molecular weight (g/mol) etc. were calculated with the OLIGO ANALYSIS TOOL (Eurofins Genomics).

With the newly-designed primer, we processed a second batch of 570 samples, summing up to a total of 760 samples, with increased taxonomic and geographic coverage. All samples in the second batch were processed similarly to what is described above.

**Figure 2.**
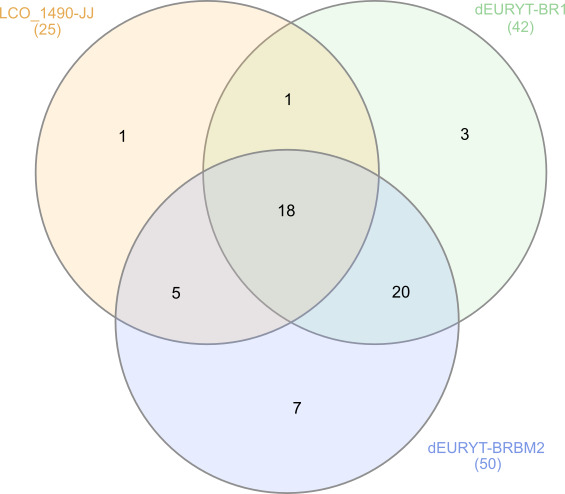
Estimated coverage of species diversity in the first batch of samples (190 specimens). Numbers in parentheses represent the number of putative species estimated with ASAP in the datasets obtained with each forward primer.

Success rates were plotted with Excel 2010 (© Microsoft 2022). To illustrate coverage of species diversity in addition to samples successfully processed, we estimated the number of putative species successfully sequenced with each forward primer and plotted them in a Venn diagram. ASAP (Puillandre et al. 2020) was used for clustering the sequences into putative species (Fig. 2).

## Results and discussion

**Figure 3.**
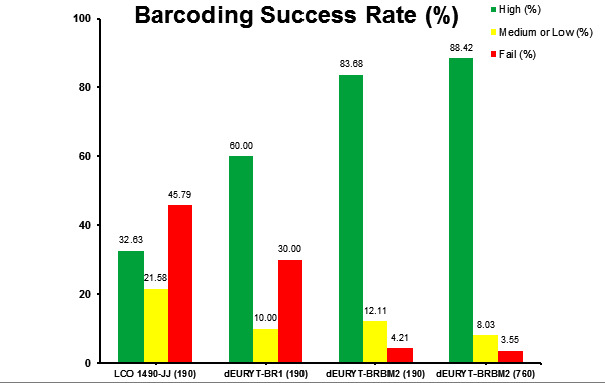
Barcoding success rates of the different forward primers. High quality means the consensus sequence is classified as quality level High by Geneious and disagreements and ambiguities within the consensus sequence are ≤ 1%. Medium or Low quality means consensus sequences are classified as such by Geneious, even if the disagreements and ambiguities are > 1%. Fail means no sequencing at all. The numbers in parentheses after the primer names show the number of included samples.

**Figure 4.**
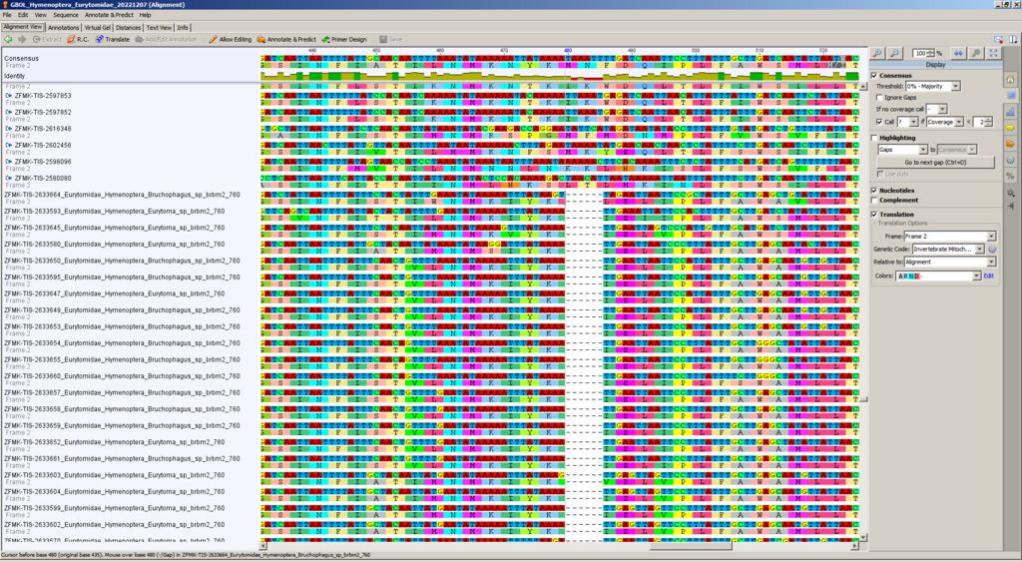
Screen shot of alignment of COI barcode of the 672 high-quality eurytomid wasps together with another 1520 Hymenoptera from GBOL. The deletion is spanning from position 480 to 485 of the standard barcode region.

Sequencing of the first 190 samples with standard primer pair LCO-JJ/HCO-JJ resulted in 32.63% high quality, 21.58% medium or low quality and 45.79% failed sequencing (for definitions of high, medium and low quality and failed barcodes, see Material and Methods section and caption of Fig. 3). Repeating the 190 samples with the intermediate forward primer dEURYT-BR1 already increased the success rate significantly (fragment length 609 bp) (Fig. 3), but still, the proportion of failed sequencing was unsatisfyingly high. Using the again optimised forward primer dEURYT-BRBM2 (in combination with the standard reverse primer HCO 2198-JJ), we received 83.68% high quality DNA barcodes (fragment length 607 bp), adding some of medium or low quality and only < 5% fails (Fig. 3). In the next step, we continued testing our new primer with a total of 760 samples, resulting in an even higher success rate of 88.4% high quality barcodes (Fig. 3). This success rate is comparable to or surpassing successfully barcoded hymenopteran taxa like Symphyta (Schmidt et al. 2016) and Apoidea (Schmidt et al. 2015). When analysing the numbers of putative species successfully barcoded with each of the primers, we see that the increased success rate with the intermediate and the new primer also represents a strong increase in covered species diversity (Fig. 2). However, Fig. 2 also shows that a total of four putative species were only successfully barcoded with the LCO-JJ or the dEURYT-BR1 forward primer, respectively. Ergo, in some cases it might be worthwhile to sequence samples at hand with a combination of primers to fully capture species diversity. It is worth noting here that the DNA barcode region shows a two triplets deletion unique to the Eurytomidae (Fig. 4, Suppl. material 4). Deletions in the CO1 gene are known from other taxonomic groups (Park et al. 2010), but have not been reported from Chalcidoidea so far.

In summary, with the new primer, it is now possible for the first time to DNA barcode Eurytomidae at an acceptable success rate. This opens up a number of possibilities: a) including Eurytomidae in the DNA barcode reference libraries, b) including Eurytomidae in (meta-)barcoding-based studies in, for example, biodiversity monitoring, conservation or ecology and c) using DNA barcodes in integrative taxonomy studies on Eurytomidae. All points are obviously linked, with taxonomically clarified and named species added into the reference databases and species-specific information included and analysed in subsequent biodiversity studies.

Being able to successfully barcode Eurytomidae is particularly relevant because Eurytomidae are very common in Central European terrestrial ecosystems. We found them in many, sometimes in high numbers, of the samples studied, with samples originating from various regions of Germany and being collected at different times of the year, with different methods. Further considering their diverse interactions with numerous insect and plant taxa, this result highlights the key role that eurytomids play and that they need to be considered and included in biodiversity studies. A widely similar situation has been recently reported and widely solved by proposing a dedicated lab protocol and primers for the widespread parasitoid wasp superfamily Ceraphronoidea (Vasilita et al. 2022).

It becomes apparent that (meta-)barcoding-based biodiversity studies will need to routinely use different primers to prevent severe biases, severe gaps and, eventually, possible severe misconceptions due to non-randomly excluded taxonomic groups and ecological guilds (for example, parasitoids). Alternatively, the use of primer-free shotgun metagenomics approaches to gain DNA barcode data should be extended, especially when protocols become more robust and costs decrease (Crampton-Platt et al. 2016, Garrido‐Sanz et al. 2022). Simultaneously, to increase the taxonomic clarity and completeness of barcode reference databases, the DNA barcodes that we are now able to generate can be used in a doubled function, first serving as one source of evidence in species delimitations, following the unified species concept (De Queiroz 2007) in an integrative taxonomy framework and second, serving as the actual database entries, linked to accessible voucher specimens, Biobank DNA repositories and to life history and distribution data. In summary, the possibility of DNA barcoding all relevant taxa at high success rates will help to transform “Dark Taxa” into known taxa, illuminate biodiversity and substantially improve the scope and quality of (meta-) barcoding-based biodiversity research.

## Supplementary Material

B4D52400-8186-5584-8BCA-B733537AD300 10.3897/BDJ.11.e101998.suppl1 7923803 Supplementary material 1 The binding site of different forward primers Data type Alignment File: oo_784349.fas https://binary.pensoft.net/file/784349 Björn Müller, Björn Rulik

F135D55C-1417-5FD9-B956-0C6AA126BD05 10.3897/BDJ.11.e101998.suppl2 Supplementary material 2 Alignment fo the primer dEURYT-BR1 Data type Alignment File: oo_784419.fas https://binary.pensoft.net/file/784419 Björn Müller, Björn Rulik

B492D67D-06F3-5003-B9EC-4AB26E754B43 10.3897/BDJ.11.e101998.suppl3 Supplementary material 3 Alignment for the primer dEURYT-BRBM2 Data type Alignment File: oo_784420.fas https://binary.pensoft.net/file/784420 Björn Müller, Björn Rulik

5FA15158-4B43-57CE-9AD9-5A79F3CAF887 10.3897/BDJ.11.e101998.suppl4 Supplementary material 4 Alignment of COI barcode Data type Alignment Brief description Alignment of COI barcode of the 672 high quality eurytomid wasps together with another 1520 Hymenoptera from GBOL. The deletion is spanning from position 480 to 485 of the standard barcode region. File: oo_784347.fasta https://binary.pensoft.net/file/784347 Björn Müller, Björn Rulik

## Figures and Tables

**Table 1.** Primers used for amplification.

Primer	Direction	Sequence length	Primer sequence	Reference
LCO 1490-JJ	F	20	5’CHACWAAYCATAAAGATATYGG 3’	Astrin & Stüben (2008)
HCO 2198-JJ	R	25	5‘AWACTTCVGGRTGVCCAAARAATCA 3‘	Astrin & Stüben (2008)
dEURYT-BR1	F	24	5‘GGWATATGAGCWGGADTTTTDGGW 3‘	herein
dEURYT-BRBM2	F	26	5’GGWATATGAGCWGGADTTTTDGGWYT 3’	herein
